# Plasma Electrolytic Polishing of Nitinol: Investigation of Functional Properties

**DOI:** 10.3390/ma14216450

**Published:** 2021-10-27

**Authors:** Kristina Navickaitė, Lucia Ianniciello, Jaka Tušek, Kurt Engelbrecht, Christian R. H. Bahl, Michael Penzel, Klaus Nestler, Falko Böttger-Hiller, Henning Zeidler

**Affiliations:** 1 Institute for Machine Elements, Engineering Design and Manufacturing, Technical University Bergakademie Freiberg, Chair for Additive Manufacturing, Agricolastrasse 1, 09599 Freiberg, Germany; michael.penzel@imkf.tu-freiberg.de (M.P.); henning.zeidler@imkf.tu-freiberg.de (H.Z.); 2 Beckmann-Institut für Technologieentwicklung e.V., Annaberger Str. 73, 09111 Chemnitz, Germany; nestler@beckmann-institut.de (K.N.); boettger-hiller@beckmann-institut.de (F.B.-H.); 3 Department of Energy Conversion and Storage, Technical University of Denmark, Anker Engelunds Vej, 2800 Kongens Lyngby, Denmark; luciann@dtu.dk (L.I.); kuen@dtu.dk (K.E.); chrb@dtu.dk (C.R.H.B.); 4 Faculty of Mechanical Engineering, University of Ljubljana, Aškerceva 6, SI-1000 Ljubljana, Slovenia; jaka.tusek@fs.uni-lj.si

**Keywords:** plasma electrolytic polishing (PeP), shape memory alloys, nitinol, mechanical stability, elastocalorics, medical applications

## Abstract

A novel, environmentally friendly, fast, and flexible polishing process for Nitinol parts is presented in this study. Nitinol samples with both superelastic and shape memory properties at room temperature were investigated. The chemical contamination and surface roughness of superelastic Nitinol plates were examined before and after plasma electrolytic polishing. The shift in phase transformation temperature and tensile strength before and after the polishing process were analysed using Nitinol wire with shape memory properties. The obtained experimental results were compared to the data obtained on reference samples examined in the as-received condition. It was found that plasma electrolytic polishing, when the right process parameters are applied, is capable of delivering Nitinol parts with extremely high surface quality. Moreover, it was experimentally proven that plasma electrolytic polishing does not have a negative impact on functionality or mechanical properties of polished parts.

## 1. Introduction

Although the shape memory effect was already known in Au-Cd and Cu-Zn alloys [1], the discovery of the superelastic and shape memory alloy (SMA) Nitinol caused significant changes in the industrial world. Nitinol has found success in a wide range of engineering applications from mobile phone antennas and heat engines to medical applications, e.g., stents and dental devices [1,2,3,4]. Moreover, nearly-equiatomic Ni-Ti is the most widely exploited alloy for elastocaloric cooling applications [5,6,7,8,9,10,11,12,13,14].

Elastocaloric applications as well as medical devices exploiting the superelasticity and shape memory of Nitinol require complex geometries with high surface quality [1,9,15,16]. In elastocaloric cooling applications, high surface roughness is associated with a low structural fatigue resistance, since small surface defects act as crack nucleation sites [8]. These cracks evolve during the cycling [9] and finally lead to failure of the part. It was demonstrated by Engelbrecht et al. (2016) [17] that by polishing rough edges of thin elastocaloric regenerator elements, their fatigue resistance increases significantly under tensile loading. Low surface roughness is preferred in medical applications because the wettability of smooth surfaces is higher compared to rough ones, improving cell adherence [18], i.e., a bone and an implant develop a bone-implant composite more easily. However, the adhesion rate of different types of cells was shown to vary depending on the surface roughness of Nitinol plates [19].

**Figure 1:**
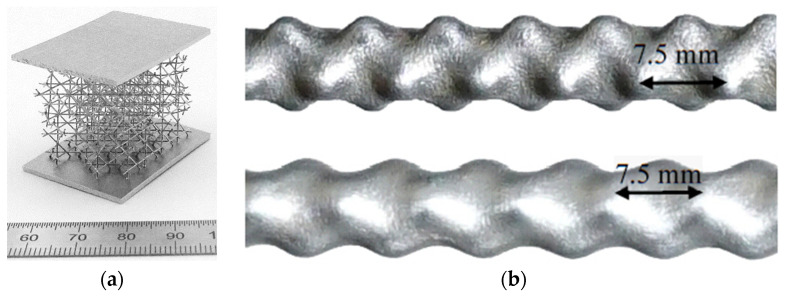
An example of an additively manufactured (**a**) stainless steel structure designed and manufactured at IFW Dresden and PeP at Beckmann Institute for Technology Development and (**b**) aluminium double corrugated tubes [28].

Nevertheless, most of the conventional polishing techniques, e.g., mechanical polishing, are unsuitable for polishing complex geometries, as shown in Figure 1. Other methods, such as electrochemical polishing (EP) or etching, use strong, hazardous and toxic acids, such as Nitric acid, hydrofluoric acid and n-Butanol, that cannot be reused [20,21,22,23,24]. Even though it has been demonstrated that chemical etching could be successfully used as a post-treatment technology even for additive manufactured Nitinol parts [16], it does require strong acidic solutions. Moreover, it was demonstrated that chemical polishing causes chemical contamination of the treated surfaces [25]. This hinders the performance of the polished parts since these chemical impurities affect the phase transformation behaviour [26] and act as crack nucleation sites [7,9]. Sandblasting, on the other hand, is used to polish complex geometries, as shown in Figure 1b. However, this produces a large amount of dust and gradually destroys nozzles supplying sand [27]. Thus, a new method of polishing complex geometries, especially of functional materials, is required.

**Figure 2:**
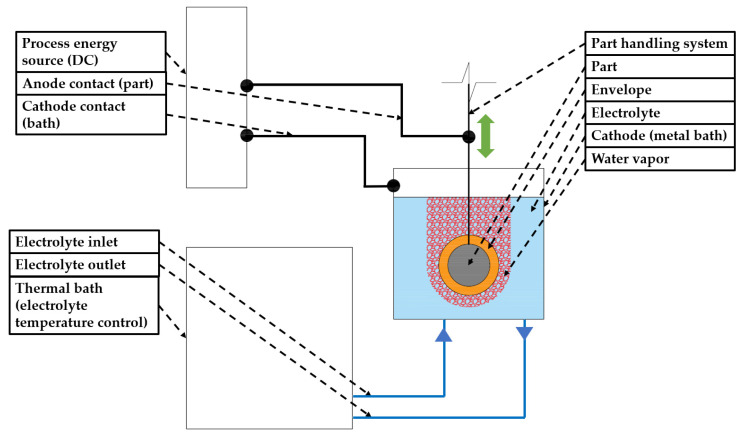
A schematic of the plasma electrolytic polishing process.

A novel polishing technique, called plasma electrolytic polishing (PeP), was introduced as an environmentally friendly alternative to conventional surface polishing techniques [29,30,31]. Further advantages of PeP are the sterilisation of polished surfaces [32] as well as achievable low surface roughness [21,32,33,34]. Moreover, PeP allows for achieving the desired surface quality in a significantly shorter process time compared to other polishing techniques. It was also demonstrated that PeP is capable to remove a wear-resistant coating from stainless chromium steel and simultaneously provide surface polishing [30]. The PeP technology uses a chemically neutral, non-toxic, water-based weak salt, i.e., ammonium sulphate [35] solution, as an electrolyte [20,29]. The chemical composition of the used electrolyte is work material specific. As the PeP name implies, only electrically conductive materials can be polished by this technique. As one can see in Figure 2, which presents a principal scheme of PeP, a workpiece, acting as an anode, is submerged in an electrolyte bath, which acts as a cathode, and DC voltage is applied. Typically, the supplied voltage could vary from 180 V to around 400 V, which is necessary to obtain a stable plasma envelope ensuring process stability [36]. In principle, one could draw similarities between PeP and EP, i.e., both processes use DC voltage to induce an electrochemical metal dissolution process in an electrolyte bath. Nevertheless, few significant differences exist between the two processes. As mentioned above, chemically neutral water-based salt solution is used for the PeP process instead of strong acids used for EP. As will be discussed further, to obtain a stable PeP process, electrolyte temperature should be in the range of 50–90 °C, while for the EP process temperatures in a range from 0–55 °C have been reported [23,37]. On the other hand, the required current density for the PeP process is significantly higher than for EP, which is ca. 0.33 A cm^−2^ [31] and in between 0.04 A cm^−2^ and 0.08 A cm^−2^ [37], respectively. Finally, PeP provides the desirable results in a considerably shorter time compared to EP [31,37]. Thus, the main differences between PeP and EC are the (non)harmfulness of electrolytes used, required electrolyte temperature, applied current density, and process speed.

From Figure 2, it is clear that the outer surface is polished during PeP. However, it was experimentally demonstrated that, using a specially designed PeP device, polishing of inner surfaces of medium-carrying pipes is also possible [33].

During the PeP process, a highly electrically conductive plasma layer, often referred to as plasma skin or plasma envelope, is formed around the part. Electric discharge between the part and electrolyte takes place, resulting in the reduction of the highest peaks of surface roughness [33,36,38]. The exact mechanism of surface smoothening during PeP is still debatable. However, the main hypotheses suggest that the peaks of surface roughness are melted or dissolved. These hypotheses are further described in the review article by Belkin et al. [21]. Parfenov et al. [34] claim that PeP is a predominantly electrochemical process during which the anodic water electrolysis reaction is shifted to a metal dissolution reaction providing surface smoothening. At the end, final surface roughness of the polished workpiece depends on the polishing time, initial roughness, electrolyte temperature, applied voltage, etc. [21]. It is worth noting that desirable surface roughnesses can be obtained even for parts with relatively high surface roughness by applying PeP for a sufficiently long time. This was demonstrated on additive manufactured (AM) Nitinol smart springs, which were polished for 20 min [39,40]. The mechanical properties of the AM and PeP springs were analysed and compared to those of conventionally manufactured Nitinol smart springs. It was reported that the phase transformation temperature of AM and PeP springs differs from the conventional ones. However, this change is mainly attributed to the AM process, since nickel powder, having a lower evaporation temperature compared to titanium, partially evaporated during the laser beam melting (LBM) process [41,42,43,44], causing tremendous changes in the transformation temperature of the manufactured parts. On the other hand, titanium is highly reactive and tends to form various precipitates, also affecting the transformation temperature as well as the homogeneity of the produced parts [41,42,43,45].

One can argue that by applying such a high voltage as required for a successful PeP process, the temperature of the workpiece would be exceedingly high, and thus could negatively affect properties of functional materials such as Nitinol. It was experimentally demonstrated that the stability of a PeP process as well as the temperature of a part strongly depends on the initial temperature of the electrolyte [21,35]. This is because the temperature of the electrolyte affects the stability of plasma layer as well as the electric conductivity, viscosity, and surface tension of an electrolyte [21]. It must be emphasised that a low electrolyte temperature, i.e., lower than 50 °C, leads to a so-called heating mode of a part rather than polishing mode [35]. On the other hand, some materials, such as steel 08Cr18Ni10Ti, could be polished when the temperature of an electrolyte is around 35 °C or 40 °C. Nevertheless, additional hydrochloric acid should be used in order to achieve a stable PeP process [21]. It was also reported that during the polishing mode, the main heat energy source is actually a plasma layer, since it presents the part of the electric chain with the highest electrical resistivity [21]. Thus, the temperature of a part cannot exceed the boiling point of an electrolyte. Despite that fact, certain materials, such as Nitinol and other SMA alloys, might be susceptible to temperatures that a part would encounter during PeP. Therefore, it should be thoroughly investigated how PeP would affect the mechanical and functional properties of materials such as Nitinol.

This study investigates the changes of the surface roughness, phase transformation temperatures, mechanical properties, and chemical composition of Nitinol wire and flat plates before and after PeP. For this purpose, a thin, cold drawn Nitinol wire with diameter *d* of 1 mm and cold rolled thin plates with thickness *σ* of 0.25 mm were acquired. It must be emphasised that the investigated Nitinol wire exhibits a shape memory effect at room temperature, while the analysed plates are superelastic. The samples were polished for several different time spans in as-received conditions as well as after taking a shape setting step. Properties of the polished samples were compared to the reference samples in as-received conditions.

## 2. Materials and Methods

### Nitinol Samples

**Figure 3:**
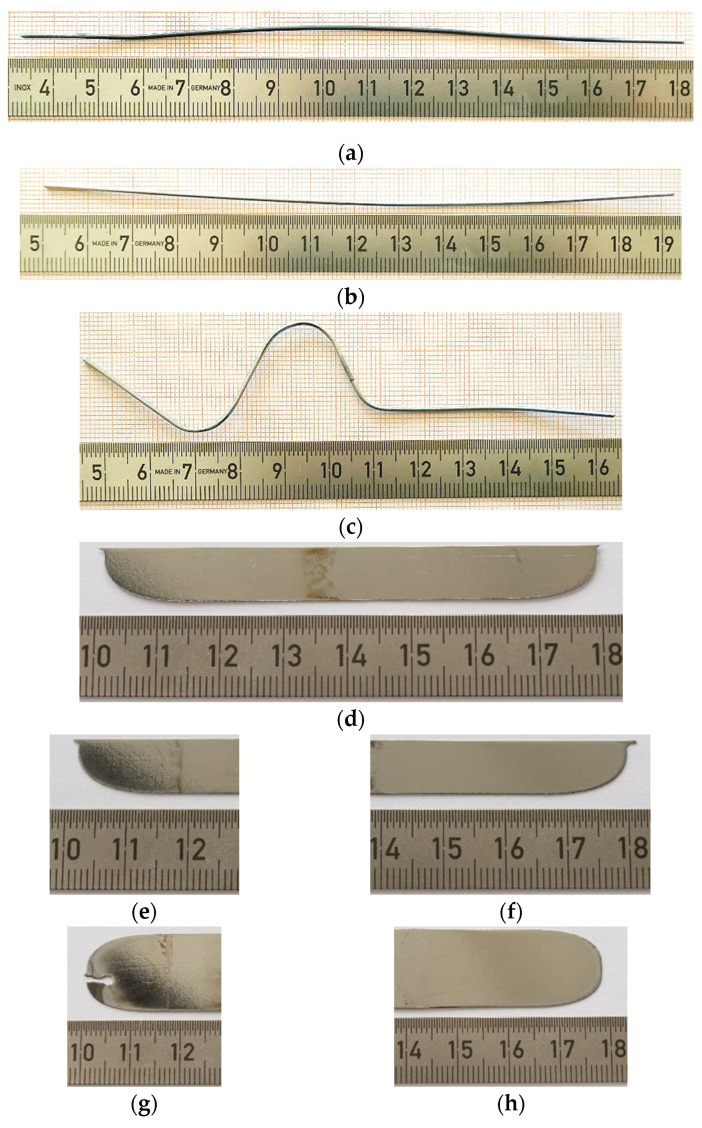
Investigated Nitinol samples (**a**) Wire 1 in the as-received condition, (**b**) Wire 2 after 120 s of PeP, (**c**) Wire 3 after shape setting step and 120 s of PeP, (**d**) Plate 1 after PeP, (**e**) Plate 2_1 after PeP, (**f**) Plate 2_2 after PeP, (**g**) Plate 3_1 after PeP and (**h**) Plate 3_2 after PeP.

In total, eight wire and three flat plate samples, shown in Figure 3, were investigated in this study. The geometrical characteristics, material properties, and the performed sample treatments as well as PeP conditions are listed in Table 1. The wire samples were cut from the same wire batch using pliers. The plate samples were cut from a sheet using electric discharge machining (EDM), hence the burn mark on Plate 3_1, which served as an orientation mark for energy dispersive X-ray spectroscopy (EDX).

Surface roughness measurements as well as EDX before and after PeP were carried out only for plate samples due to the easier handling and possibility to more precisely locate positions of previous measurements. The differential scanning calorimetry (DSC) and mechanical tensile strength measurements were performed only on wire samples.

## 3. Results and Discussion

### 3.1. Plasma Electrolytic Polishing

For polishing the Nitinol samples, a specific water-based electrolyte was used to separately PeP each sample. The plate samples were exposed to different durations of PeP treatment in order to determine the minimum necessary time for reducing the surface roughness on sample edges down to the surface roughness values of the frontal surface. Since there was no information concerning whether PeP causes any chemical contamination of Nitinol samples, and if the contamination level depended on the exposure time, each plate was polished in two steps, i.e., one end of a single plate was polished for a longer/shorter time than the other end of the same plate.

The wire samples were polished in as-received condition (Wire 2, Wire 5, and Wire 8) as well as after the shape setting step (Wire 3 and Wire 6), which was carried out in a furnace at 520 °C for 10 min followed by rapid quenching in a bath filled with cold tap water as recommended by most manufactures and literature [4,46]. With the purpose to investigate whether the Nitinol wires would show a shape memory effect (SME) during PeP, i.e., if the temperature of the sample exceeds the austenite finish temperature, *A*_f_, during the PeP process, as well was whether additionally introduced stress, i.e., restriction of the deformation, would have an influence on the SME of the material and its transformation temperature after PeP, Wire 3 and Wire 6 were used. Note that all wires used in this study had a black oxide layer in as-received conditions. This oxide layer was cleaned off only from the tips of each wire for improving electrical contact for the PeP process.

First, Wire 3 was fixed at one end by firmly clamping it to the anode contact, while the other end and the rest of the wire were allowed to deform/move freely. Subsequently, Wire 6 was firmly clamped at both ends so that during the PeP process it could not deform/move at any point. As mentioned above, the restriction to assume the pre-set shape, i.e., firm clamping, might induce internal stresses inside the material causing changes in its *A*_f_, which emphasises the importance of an adequate clamping system of Nitinol wires during the PeP process. Both samples were stretched into a straight line before the PeP process. Wire 3 demonstrated one-way SME during the PeP process by completely assuming its pre-set shape since the temperature of the electrolyte exceeded the *A*_f_ temperature of the wire. It is important to keep in mind that none of the polished wires received any shape memory training. Note that the optimal temperature of the electrolyte for polishing Nitinol samples was experimentally determined to be *T*_e_ = 80 °C. More specifically, the range of electrolyte temperature from *T*_e_ = 30 °C to *T*_e_ = 90 °C with the step of 10 °C was investigated on as-received test wire for polishing time of 60 s. The look of the surface of each polished test wire was visually evaluated for removal of oxide layers, burn marks and shininess of the surface. Since the best results were obtained when electrolyte temperature was *T*_e_ = 80 °C, it was chosen for further experiments. The applied voltage and electrolyte temperature was constant for each polished sample. However, due to the slightly different mass and surface area of each sample, slight variations in effectively applied voltage occurred.

### 3.2. Surface Roughness Measurements

**Figure 4:**
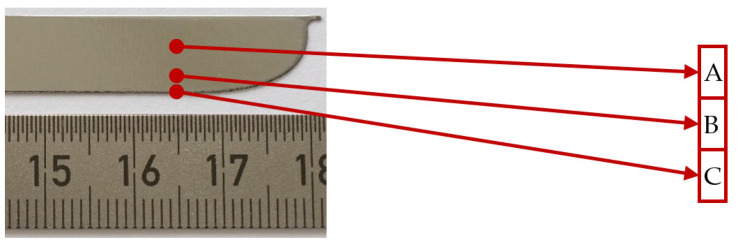
The position for surface roughness measurements shown on Plate 2_2.

Surface roughness measurements were performed only on the plate samples using a Keyence VK-9700 scanning laser microscope with maximal *z*-axis resolution of 1 nm according to the ISO 4287 standard. The measurements were performed in the middle of a plate (position A), right above the cutting edge (position B) and directly on it (position C), as shown in Figure 4. The results of the surface roughness measurements before and after PeP are given in Table 2. Note that before PeP, the surface roughness was measured at the centre of each plate considering it as a characteristic location at the positions A, B, and C. Due to the different polishing time of each side of Plate 2 and Plate 3, the surface roughness measurements after PeP were carried out at the end of each plate side, as shown in Figure 4.

**Figure 5:**
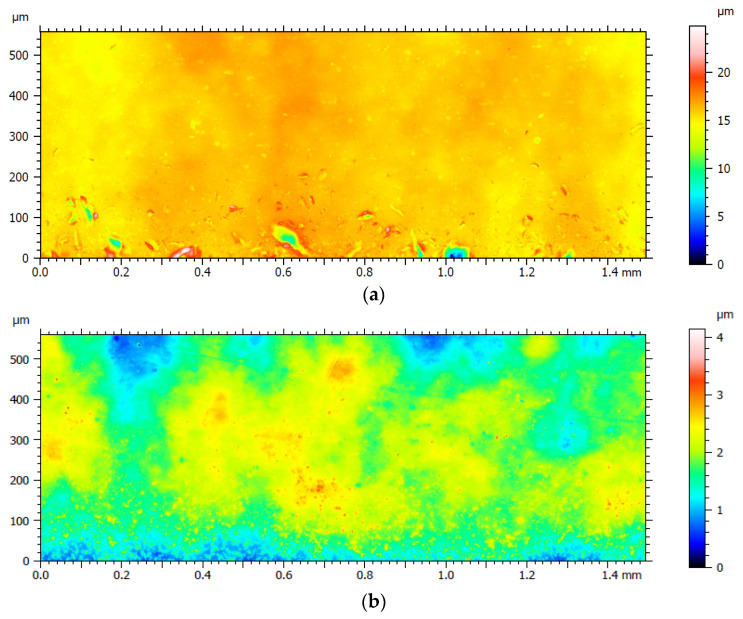
Surface roughness measurement of Plate 3_1 (**a**) before and (**b**) after PeP. Note the different *z* scale bar.

One can see from Table 2 that the surface roughness of Nitinol Plate 3_1 is reduced already after *t* = 120 s of PeP from *Ra*_C = 0.85 μm to *Ra*_C = 0.17 μm at the cutting edge. Further polishing resulted in lower surface roughness not only on the cutting edge of the plate but also on the middle part of it. As explained above, the electric field lines during PeP are focusing on the highest peaks of the surface, resulting in their dissolution/melting [21] and allowing sufficient time for the PeP process to take place, even very comparatively high surface roughnesses could be smoothened out [39]. Thus, after at least *t* = 120 s of PeP, significantly lower surface roughness of the polished samples was achieved. However, it is noticeable that the surface roughness of the cutting edge of the plates polished shorted than *t* = 120 s was increased. This could be due to too short a polishing time, when only an old oxide layer was removed exposing the plain material and leaving small cavities caused by the electric discharge on it. Similar observations were also made during experiments carried out by Nevyantseva et al. [30]. Figure 5 presents a characteristic surface roughness measurement area before and after PeP, from which the results of measurement locations B and C were obtained, for Plate 3_1, and the full data are provided in Mendeley Data at DOI: 10.17632/rwnn9w3993.1.

### 3.3. EDX Measurements

The plate samples were cleaned with ethanol and kept in vacuum atmosphere for at least six hours before the energy dispersive X-ray spectroscopy (EDX) measurements. Each plate was measured at six equally distributed points. Three measurements were taken at each measurement location for the statistical analysis. The measurements were carried out using a scanning electron microscope ZEISS LEO1455VP with X-ray micro-area analysis EDAX GENESIS.

After comparing the results of EDX measurements of the plates before and after PeP, no significant difference in material composition was found, as shown in Table 3. The measured slight deviation of the chemical composition could be attributed to the measurement uncertainty, which is 2 wt% for main components. There was also no chemical contamination of the plate surface found, indicating that PeP does not influence the chemical composition of polished Nitinol parts.

**Figure 6:**
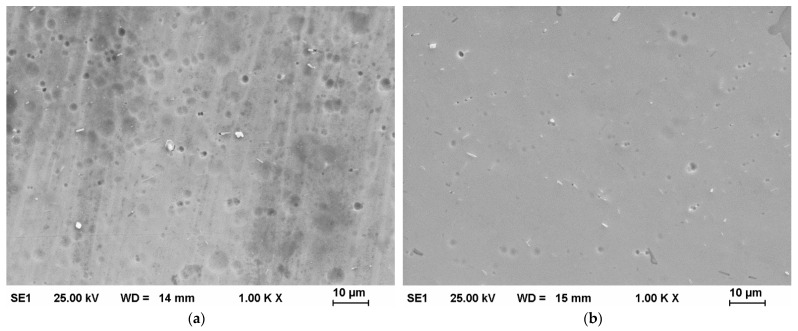
Scanning electron microscopy pictures of Plate 3_2 (**a**) before PeP and (**b**) after PeP.

In Figure 6a, one can see that initially the surface of Plate 3_2 contained circular randomly distributed surface relief which could have been caused by debris on tools during the cold rolling process. This pattern was almost completely removed after *t* = 180 s of PeP. However, small surface defects were uncovered, as can be observed in Figure 6b.

### 3.4. DSC Measurements

The DSC measurements were performed using a NETZSCH DSC 200 F3 apparatus under an Ar atmosphere. The temperature ramp rate was 1 K/min and the temperature range of the measurements was from −50 to 200 °C. The measurements were started with the heating cycle for all the samples. The samples were cut using pliers and at least 0.5 mm material was ground away from each side to remove any remanence of plasticly deformed material. Finally, the entire surface of each sample was ground using sandpaper with grit P2500 before measurements to remove the formed oxide layer. One can argue that subsequent grinding of the surface of the DSC samples abolishes the attained PeP effect. This is true for the surface roughness, i.e., grinding samples with the sandpaper increases surface roughness, *Ra*. However, in the case of PeP affecting the phase transformation temperature of the bulk Nitinol part, a careful grinding of the oxide layer using fine sandpaper does not diminish the PeP effect. On the other hand, if the oxide layers are not removed immediately before DSC measurements, the obtained data would not be reliable. This is because the phase transformation temperature would be obtained not only for Nitinol, but also for the oxide layer.

**Figure 7:**
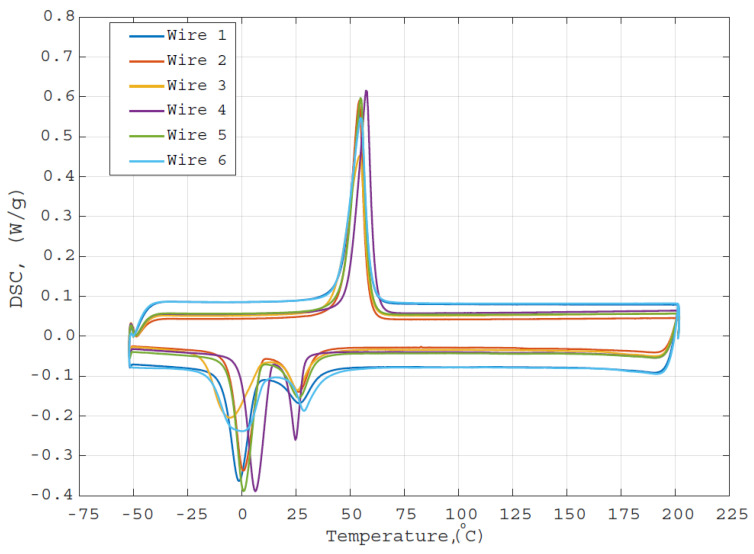
Results of DSC measurements.

Figure 7 presents the DSC results, from which it can be seen that PeP does not cause a shift in the phase transition temperature of the examined Nitinol wire samples. The slight deviation of the temperatures, observed from the measurement results, might be caused by sample inhomogeneities, slight differences in sample preparations, as well as measurement inaccuracies. However, one can note from Figure 7 that for Wire 6, which was deformed and fixed on both ends during the PeP process, the martensite-austenite peak during cooling became somehow broader and lower after PeP compared to other wires. The austenite-martensite peak during heating seems not to be influenced by the PeP process in the deformation-restricted condition though. The changes occurring in the martensite-austenite peak could be due to induced internal stresses of the material, which occurred during the polishing, since the sample was fixed, hindering its SME. Nevertheless, further investigation is needed in order to draw concrete conclusions from the obtained findings.

### 3.5. Mechanical Tensile Strength Test

**Figure 8:**
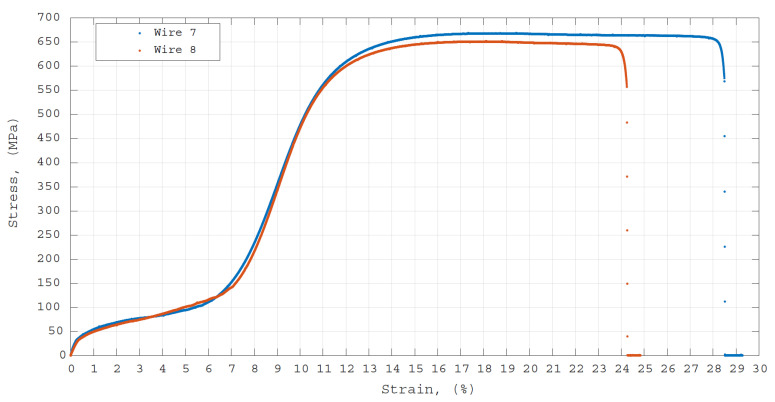
Results of the mechanical tensile stress tests as a stress—strain relation.

Mechanical tensile strength tests were carried out on Wire 7 and Wire 8. These tests were carried out at room temperature, meaning that wires were in their martensite phase, using a Zwick Z050 universal testing machine (UTM) with a 5 kN load cell at 0.09 mm/s of loading and unloading speed. The samples had a similar gauge length of 40 mm and displacement of the crosshead was recorded. The results of the mechanical tensile test are shown in Figure 8. One can see that no significant difference in the stress–strain response of Wire 7, which was tested in as-received condition, compared to Wire 8, which was polished for *t* = 120 s, was observed.

## 4. Conclusions

This study presents a novel, environmentally friendly alternative for polishing Nitinol parts for applications that require high surface quality. It was demonstrated that plasma electrolytic polishing is capable of achieving low surface roughness of samples cut using EDM without causing chemical contamination of their surfaces. It was also proven that PeP does not cause a shift in the phase transformation temperature of the polished Nitinol wires and does not affect their mechanical properties.

It was observed in this study that Nitinol parts exhibit SME during the polishing process. This could be seen as a disadvantage when parts for certain medical applications are polished, since PeP should be the last step in in the production chain of these parts in order to keep them sterilised. On the other hand, as reported in the literature, at a certain electrolyte temperature, the parts experience exceedingly high temperatures during PeP. This could be exploited as a shape setting step combined with immediate polishing. Nevertheless, this hypothesis must be experimentally validated.

Finally, findings of this study encourage further research and more thorough analysis of the functionality of Nitinol parts after PeP. As a future research prospect, the suitability of additive manufacturing technologies in combination with PeP should be investigated for producing Nitinol parts with complexed geometries that are demanded by both medical and elastocaloric applications. The influence of PeP on the hysteresis of polished Nitinol parts will be investigated as well.

## Figures and Tables

**Table 1:** Geometrical characteristics and material properties of the analysed samples.

Sample Name	Length, *l*, (mm)	Diameter/Width x Thickness, *d*, *w* x *σ*, (mm)	Austenite Finish, *A*_f_, (°C)	Shape Setting, (Y/N)	PeP Time, *t*, (s)	Voltage, *U*, (V)	Current, *I*, (A)	Electrolyte Temp., *T*_e_, (°C)
Wire 1	150	1	70 ± 5 ^1^	N	0	0	0	0
Wire 2	150	1	70 ± 5 ^1^	N	120	330	1	80
Wire 3	150	1	70 ± 5 ^1^	Y	120	330	1	80
Wire 4	150	1	70 ± 5 ^1^	Y	0	0	0	0
Wire 5	150	1	70 ± 5 ^1^	N	60	330	1	80
Wire 6	150	1	70 ± 5 ^1^	Y	120	330	1	80
Wire 7	40	1	70 ± 5 ^1^	N	0	0	0	0
Wire 8	40	1	70 ± 5 ^1^	N	120	331	3	80
Plate 1	78	90 × 0.25	0 ± 7 ^1^	N	10	335	3	80
Plate 2_1	39	90 × 0.25	0 ± 7 ^1^	N	30	334	3	80
Plate 2_2	39	90 × 0.25	0 ± 7 ^1^	N	60	334	3	80
Plate 3_1	39	180 × 0.25	0 ± 7 ^1^	N	120	334	4	80
Plate 3_2	39	180 × 0.25	0 ± 7 ^1^	N	180	333	4	80

^1^ Data provided by the retailer.

**Table 2:** Surface roughness, *Ra*, measurement results before and after PeP.

Plate Name	PeP Time, *t*, (s)	Before PeP			After PeP		
		*Ra*_A, (μm)	*Ra*_B, (μm)	*Ra*_C, (μm)	*Ra*_A, (μm)	*Ra*_B, (μm)	*Ra*_C, (μm)
Plate 1	10	0.12	0.12	0.37	0.11	0.11	0.54
Plate 2_1	30	0.11	0.13	0.17	0.11	0.13	0.30
Plate 2_2	60				0.10	0.11	0.40
Plate 3_1	120	0.15	0.13	0.85	0.08	0.08	0.17
Plate 3_2	180				0.09	0.13	0.16

**Table 3:** Chemical composition of the analysed plates obtained by EDX before and after PeP.

Sample Name	Element in at%	Before PeP	After PeP
Plate 1	Ni	51	51
	Ti	49	49
Plate 2_1	Ni	52	51
	Ti	48	49
Plate 2_2	Ni	52	51
	Ti	48	49
Plate 3_1	Ni	52	51
	Ti	48	49
Plate 3_2	Ni	52	51
	Ti	48	49

## Data Availability

The data that support the findings of this study are openly available in Mendeley Data at doi: 10.17632/rwnn9w3993.1 or on reasonable request to the corresponding author.

## References

[1] Duerig T., Pelton A., Stöckel D. (1999). An overview of nitinol medical applications. Mater. Sci. Eng. A.

[2] Kauffman G.B., Mayo I. (1997). The story of nitinol: The serendipitous discovery of the memory metal and its applications. Chem. Educ..

[3] Mwangi J.W., Nguyen L.T., Bui V.D., Berger T., Zeidler H., Schubert A. (2019). Nitinol manufacturing and micromachining: A review of processes and their suitability in processing medical-grade nitinol. J. Manuf. Process..

[4] Pelton A.R., Duerig T.W., Stöckel D. (2004). A guide to shape memory and superelasticity in Nitinol medical devices. Minim. Invasive Ther. Allied Technol..

[5] Tušek J., Žerovnik A., Čebron M., Brojan M., Žužek B., Engelbrecht K., Cadelli A. (2018). Elastocaloric effect vs fatigue life: Exploring the durability limits of Ni-Ti plates under pre-strain conditions for elastocaloric cooling. Acta Mater..

[6] Engelbrecht K. (2019). Future prospects for elastocaloric devices. J. Phys. Energy.

[7] Bruederlin F., Bumke L., Chluba C., Ossmer H., Quandt E., Kohl M. (2018). Elastocaloric cooling on the miniature scale: A review on materials and device engineering. Energy Technol..

[8] Kirsch S.-M., Welsch F., Michaelis N., Schmidt M., Wieczorek A., Frenzel J., Eggeler G., Schütze A., Seelecke S. (2018). NiTi-based elastocaloric cooling on the macroscale: From basic concepts to realization. Energy Technol..

[9] Kabirifar P., Žerovnik A., Ahčin Ž., Porenta L., Brojan M., Tušek J. (2019). Elastocaloric cooling: State-of-the-art and future challenges in designing regenerative elastocaloric devices. Stroj. Vestn. J. Mech. Eng..

[10] Tušek J., Engelbrecht K., Mañosa L., Vives E., Pryds N. (2016). Understanding the thermodynamic properties of the elastocaloric effect through experimentation and modelling. Shape Mem. Superelasticity.

[11] Nikitin S.A., Myalikgulyev G., Annaorazov M.P., Tyurin A.L., Myndyev R.W., Akopyan S.A. (1992). Giant elastocaloric effect in FeRh alloy. Phys. Lett. A.

[12] Porenta L., Kabirifar P., Žerovnik A., Čebron M., Žužek B., Dolenec M., Brojan M., Tušek J. (2020). Thin-walled Ni-Ti tubes under compression: Ideal candidates for efficient and fatigue-resistant elastocaloric cooling. Appl. Mater. Today.

[13] Ossmer H., Chluba C., Krevet B., Quandt E., Rohde M., & Kohl M. (2013). Elastocaloric cooling using shape memory alloy films. J. Phys. Conf. Ser..

[14] Slaughter J., Czernuszewicz A., Griffith L., Pecharsky V. (2020). Compact and efficient elastocaloric heat pumps—Is there a path forward?. J. Appl. Phys..

[15] Navickaitė K., Penzel M., Bahl C., Engelbrecht K., Tušek J., Martin A., Zinecker M., Schubert A. (2020). CFD-simulation assisted design of elastocaloric regenerator geometry. Sustainability.

[16] Jahadakbar A., Nematollahi M., Safaei K., Bayati P., Giri G., Dabbaghi H., Dean D., Elahinia M. (2020). Design, modeling, additive manufacturing, and polishing of stiffness-modulated porous nitinol bone fixation plates followed by thermomechanical and composition analysis. Metals.

[17] Engelbrecht K., Tušek J., Sanna S., Eriksen D., Mishin O.V., Bahl C.R.H., Pryds N. (2016). Effects of surface finish and mechanical training on Ni-Ti sheets for elastocaloric cooling. APL Mater..

[18] Hansen A.W., Beltrami L.V.R., Antonini L.M., Villarinho D.J.J., das Neves C.K., Marino C.E.B., de Fraga Malfatti C. (2015). Oxide formation on NiTi surface: Influence of the heat treatment time to achieve the shape memory. Mater. Res..

[19] Wirth C., Comte V., Lagneau C., Exbrayat P., Lissac M., Jaffrezic-Renault N., Ponsonnet L. (2005). Nitinol surface roughness modulates in vitro cell response: A comparison between fibroblasts and osteoblasts. Mater. Sci. Eng. C.

[20] Vopát T., Podhorský Š., Sahul M., Haršáni M. (2019). Cutting edge preparation of cutting tools using plasma discharges in electrolyte. J. Manuf. Process..

[21] Belkin P.N., Kusmanov S.A., Parfenov E.V. (2020). Mechanism and technological opportunity of plasma electrolytic polishing of metals and alloys surfaces. Appl. Surf. Sci. Adv..

[22] Kim J., Park J.K., Kim H.K., Unnithan A.R., Kim C.S., Park C.H. (2017). Optimization of electropolishing on NiTi alloy stents and its influence on corrosion behavior. J. Nanosci. Nanotechnol..

[23] Rokicki R., Hryniewicz T. (2008). Nitinol surface finishing by magnetoelectropolishing. Trans. Inst. Met. Finish..

[24] Barbosa F.O.G., da J.A., Gomes C.P., de Araújo M.C.P. (2008). Influence of electrochemical polishing on the mechanical properties of K3 nickel-titanium rotary instruments. J. Endod..

[25] Mwangi J.W. Micro-Electrical Discharge Machining of Nitinol for Medical Applications. Ph.D. Thesis.

[26] Zanotti C., Giuliani P., Bassani P., Zhang Z., Chrysanthou A. (2010). Comparison between the thermal properties of fully dense and porous NiTi SMAs. Intermetallics.

[27] Kashapov L.N., Kashapov N.F., Kashapov R.N., Denisov D.G. (2016). Plasma electrolytic treatment of products after selective laser melting. J. Phys. Conf. Ser..

[28] Navickaitė K. (2018). Nature-Inspired Double Corrugated Geometry for Enhanced Heat Transfer. Ph.D. Thesis.

[29] Nestler K., Böttger-Hiller F., Adamitzki W., Glowa G., Zeidler H., Schubert A. (2016). Plasma electrolytic polishing—An overview of applied technologies and current challenges to extend the polishable material range. Procedia CIRP.

[30] Nevyantseva R.R., Gorbatkov S.A., Parfenov E.V., Bybin A.A. (2001). The influence of vapor-gaseous envelope behavior on plasma electrolytic coating removal. Surf. Coatings Technol..

[31] Yerokhin A., Mukaeva V.R., Parfenov E.V., Laugel N., Matthews A. (2019). Charge transfer mechanisms underlying contact glow discharge electrolysis. Electrochim. Acta.

[32] Kröning O., Schulze H.-P., Zeidler H., Kranhold C. (2019). Das Plasma-elektrolytische Polieren von Werkstücken für den medizin- technischen Einsatz. Magdebg. Ing..

[33] Cornelsen M., Deutsch C., Seitz H. (2018). Electrolytic plasma polishing of pipe inner surfaces. Metals.

[34] Parfenov E.V., Farrakhov R.G., Mukaeva V.R., Gusarov A.V., Nevyantseva R.R., Yerokhin A. (2016). Electric field effect on surface layer removal during electrolytic plasma polishing. Surf. Coat. Technol..

[35] Kusmanov S.A., Tambovskiy I.V., Kusmanova I.A., Belkin P.N. (2019). Some features of anodic plasma electrolytic processes in aqueous solution. J. Phys. Conf. Ser..

[36] Zeidler H., Nestler K., Boettger-Hiller F., Schubert A., Previtali B., Demir Gökhan A. (2016). Surface finish machining of medical parts using plasma electrolytic polishing. Procedia CIRP.

[37] Lochyński P., Charazińska S., Łyczkowska-Widłak E., Sikora A. (2019). Electropolishing of stainless steel in laboratory and industrial scale. Metals.

[38] Vana D., Podhorsky S., Hurajt M., Hanzen V. (2013). Surface properties of the stainless steel X10 CrNi 18/10 after aplication of plasma polishing in electrolyte. Int. J. Mod. Eng. Res..

[39] Stepputat V. (2020). Influence of Plasma-Electrolytic Polishing on the Functional and Mechanical Properties of Additively Manufactured Nitinol Smart Springs. Master’s Thesis.

[40] Stepputat V.N., Zeidler H., Safranchik D., Strokin E., Böttger-Hiller F. (2021). Investigation of post-processing of additively manufactured nitinol smart springs with plasma-electrolytic polishing. Materials.

[41] Andani M., Haberland T., Walker C.J., Elahinia M. An Investigation of Effective Process Parameters on Phase Transformation Temperature of Nitinol Manufactured by Selective Laser Melting. Proceedings of the ASME 2014 Conference on Smart Materials, Adaptive Structures and Intelligent Systems.

[42] Walker J., Andani M.T., Haberland C., Elahinia M. (2014). Additive manufacturing of Nitinol shape memory alloys to overcome challenges in conventional Nitinol fabrication. ASME International Mechanical Engineering Congress and Exposition.

[43] Haberland C., Elahinia M., Walker J., Meier H. Visions, concepts and strategies for smart Nitinol actuators and complex Nitinol structures produced by additive manufacturing. Proceedings of the ASME 2013 Conference on Smart Materials, Adaptive Structures and Intelligent Systems SMASIS 2013.

[44] Bormann T., Schumacher R., Müller B., Mertmann M., De Wild M. (2012). Tailoring selective laser melting process parameters for niti implants. J. Mater. Eng. Perform..

[45] DebRoy T., Wei H.L., Zuback J.S., Mukherjee T., Elmer W.J., Milewski J.O., Beese M.A., Wilson-Heid A., Zhang W. (2018). Additive manufacturing of metallic components—Process, structure and properties. Prog. Mater. Sci..

[46] Gilbert H.B., Webster R.J. (2016). Rapid, reliable shape setting of superelastic nitinol for prototyping robots. IEEE Robot. Autom. Lett..

